# Shining a light in the black box of orphan drug pricing

**DOI:** 10.1186/1750-1172-9-62

**Published:** 2014-04-27

**Authors:** Eline Picavet, Thomas Morel, David Cassiman, Steven Simoens

**Affiliations:** 1 KU Leuven Department of Pharmaceutical and Pharmacological Sciences, Herestraat 49, PO box 521, Leuven 3000, Belgium; 2 Department of Hepatology, University Hospital Leuven, Herestraat 49, Leuven 3000, Belgium

**Keywords:** Pricing, Price setting, Orphan drugs

## Abstract

**Background:**

The pricing mechanism of orphan drugs appears arbitrary and has been referred to as a “black box”. Therefore, the aim of this study is to investigate how drug- and disease-specific variables relate to orphan drug prices. Additionally, we aim to explore if certain country-specific pricing and reimbursement policies affect the price level of orphan drugs.

**Methods:**

Annual treatment costs per indication per patient were calculated for 59 orphan drugs with a publicly available price in Belgium, the Netherlands, Czech Republic, France, Italy and the United Kingdom. A multiple linear regression model was built with 14 drug- and disease-specific variables. A Mann-Whitney *U* test was used to explore whether there is a correlation between annual treatment costs of orphan drugs across countries with different pricing and reimbursement policies.

**Results:**

Repurposed orphan drugs, orally administered orphan drugs or orphan drugs for which an alternative treatment is available are associated with lower annual treatment costs. Orphan drugs with multiple orphan indications, for chronic treatments or for which an improvement in overall survival or quality-of-life has been demonstrated, are associated with higher annual treatment costs. No association was found between annual treatments cost of orphan drugs across countries and the different pricing and reimbursement systems.

**Conclusions:**

This study has shown that prices of orphan drugs are influenced by factors such as the availability of an alternative drug treatment, repurposing, etc. Current debate about the affordability of orphan drugs highlights the need for more transparency in orphan drug price setting.

## Background

Recently, the price of some orphan drugs has become an issue of much debate. For example, eculizumab, for the treatment of paroxysmal nocturnal hemoglobinuria, can cost up to US$ 500,000 per patient per year [1]. A few orphan drugs even qualify as blockbusters, as their global annual sales exceed a billion US$ [2]. Today, third-party payers are increasingly cost-conscious and concerned with escalating health care costs [1]. A Belgian study showed that the impact of orphan drugs is substantial (i.e. amounting to 1.9% of pharmaceutical expenditure in 2008) and likely to rise significantly in the future [3]. An analysis by Schey *et al*. predicted that the expenditure on orphan drugs, as a proportion of total pharmaceutical expenditure, is likely to plateau between four and five percent [4]. Given current budgetary constraints and concerns about affordability, it is likely that the price and price setting of expensive medicines, such as orphan drugs, will become the focus of attention.

Not much is known about the pricing of orphan drugs, the pricing mechanism has even been referred to as a “black box” [5,6]. Pricing of orphan drugs is unique because R & D costs need to be recouped from a small number of patients, also, orphan drugs benefit from a period of market exclusivity and there are few alternatives available [7]. Today, only a few descriptive studies have identified a number of individual factors driving orphan drug price setting. For example, an inverse correlation between the price per capita of an orphan drug and the prevalence of the indication has been reported in the literature. The rarer the indication, the more expensive the treatment is [7,8]. In contrast, production costs, molecular complexity and therapeutic benefit have not been found to correlate with the price of an orphan drug [6]. A recent study regrouped orphan drugs in four categories, i.e. first-to-market non-oncology orphan drugs, second-to-market non-oncology orphan drugs, repurposed non-oncology drugs and oncology orphan drugs. The annual weighted treatment costs of drugs in the first category, accounting for 44% of all orphan drugs, are in the highest range, with an average cost of € 200,000 per patient per year. Mean annual treatment costs of drugs in the other categories range between €16,000 and €35,000 [9].

To the best of the authors’ knowledge, no recent studies have investigated how annual treatment costs relate to different drug-, disease- and country-specific variables. The use of annual treatment costs has become an accepted method to compare prices. For example, Messori *et al*. used yearly cost per patient to define the inverse relation between price and prevalence [8]. Orofino *et al*. calculated mean annual treatment cost per patient to estimate spending on orphan drugs in several European countries [10]. Aballéa *et al*. found that low disease prevalence and low number of available therapeutic alternatives are associated with a higher annual treatment cost. However, no significant association was found between any other disease-related and drug-related variables and yearly treatment cost, possibly due to a lack of power [11]. Prices for orphan drugs also vary across countries due to different pricing and reimbursement policies (e.g. free vs. fixed pricing) [12]. Although national pricing mechanisms are often not specific to orphan drugs, they do tend to favor orphan drugs, for example, by setting up special funds for reimbursement [5,13].

The aim of this study is to investigate how various drug- and disease-specific variables relate to annual treatment costs per patient and per orphan drug indication in six European countries. Additionally, we aim to explore if certain country-specific pricing and reimbursement policies affect the price level of orphan drugs.

## Methods

All orphan drugs, listed as authorized on the website of the European Medicines Agency on April 17 2013 (n = 65) and for which a price was publicly available in Belgium, the Netherlands, Czech Republic, France, Italy or the United Kingdom were included in the analysis [14].

### Annual treatment dose

The annual treatment dose was calculated per indication according to the standard treatment plan described in the Summary of Product Characteristics (SmPC). If a starting dose was adjusted based on (unknown) performance results (e.g. for mecasermin (Increlex®)), the initial starting dose was maintained. If a dose range was given (e.g. for romiplostim (Nplate®)), then an average dose between the minimum and maximum dose was assumed. No dose adjustments were carried out to account for liver and/or kidney impaired patients. To determine the weight or Body Surface Area (BSA) necessary to calculate the dose of weight-adjusted and BSA-adjusted treatments, weight and/or height information from pivotal studies as described in the European Public Assessment Report (EPAR) were consulted at first. Secondly, patient registries (e.g. from the European Organisation for Research and Treatment of Cancer) or relevant studies, as described on clinicaltrials.gov or in publications were consulted. As a last resort, weight and/or height data was derived from standardized growth curves [15,16]. BSA was determined using the Haycock formula, a validated method for calculating BSA in infants, children and adults [17]. The annual treatment dose for children was calculated conservatively, by assuming the lowest age (in accordance with the registered indication) and no weight and/or height increase over the course of a year.

Treatment duration of 365 days was assumed, unless stated differently in the SmPC. For treatments given in cycles, the total number of cycles was based on the treatment plan. In case of varying numbers of cycles, the mean number of cycles in the main clinical study (as described in the EPAR) was assumed. In case of incatibant (Firazyr®), the number of cycles was calculated based on the chance distributions described in the EPAR. In the absence of data on the number of cycles, the number of cycles was derived from the Overall Survival (OS) time (e.g. for temsirolimus (Torisel®)).

### Annual treatment cost

Orphan drug prices were analysed in six European countries (Belgium, the Netherlands, Czech Republic, France, Italy and the United Kingdom), which were selected based on public availability of orphan drug prices (Table 1). Prices in Czech koruna (CZK) and British pound sterling (GBP) were converted into Euro by applying the exchange rates of respectively €1 = 25.95 CZK (July 2013) and €1 = 0.83 GBP (October 2013) [18]. The gross domestic products (GDP) per capita in purchasing power standard (PPS) of these countries are of similar magnitude, therefore allowing international comparison (Table 1) [19]. The EU28 index equals 100 (standard deviation = 41.33) and varies from 47 in Bulgaria to 263 in Luxemburg.

**Table 1:** Pricing data per country

	**GDP per capita in PPS**	**Source of price data**		**Date of data extraction**	**Number of orphan drugs**	Type of price*	**Distribution channel**[20]
Belgium	120	Belgian Centre for Pharmacotherapeutic Information	[21]	17/04/2013	41	Public prices	Hospital pharmacies
Netherlands	128	College voor Zorgverzekeringen	[22]	26/07/2013	40	Consumer reimbursement prices	Hospital and community pharmacies
Czech Republic	81	SÚKL State Institute for Drug Control	[23]	29/07/2013	29	Rough prices for final consumer	Hospital pharmacies
France	109	Thériaque	[24]	25/07/2013	43	Mixed prices: taxes excluded and taxes included	Hospital pharmacies
Italy	101	Informazioni Sui Farmaci	[25]	30/07/2013	21	Ex-factory prices	Hospital and community pharmacies
		Agenzia Italiana del Farmaco	[26]	02/08/2013		Maximum prices	
United Kingdom	106	British National Formulary 2013	[27]	02/10/2013	50	Net cost	Hospital pharmacies

GDP: gross domestic product, PPS: purchasing power standard, *type of prices: as described by data source: “public prices”, “consumer reimbursement prices”, “rough prices for final consumer” and “maximum prices” represent ex-factory prices increased by a distribution margin for the wholesaler, a distribution margin for the pharmacist, taxes and honoraria. “Ex-factory prices” and “net cost” are ex-factory prices without taxes and margins for distributers and pharmacists [28]. Due to differences in margins, taxes and negotiations, pricing of orphan drugs may differ dependent on the distribution channel.

Annual treatment costs (ATX) were calculated per patient per orphan drug indication and per country based on the annual treatment dose and the most recent price in each country. Vial wastage was assumed. If there was an unfinished pack at the end of the year, only a proportion of the price of that pack was accounted for. In case of multiple packaging sizes, the cheapest pack (or a combination of packs) was selected. Annual treatment costs of various formulations of the same orphan drug were calculated separately. No official prices were found for ivacaftor (Kalydeco®), alipogene tiparvovec (Glybera®), concentrate of proteolytic enzymes enriched in bromelain (NexoBrid®) and teduglutide (Revestive®) in any of the six countries. Additionally, we were unable to calculate the annual treatment cost for hydroxycarbamide (Siklos®) and sapropterin dihydrochloride (Kuvan®) due to the patient-specific nature of those treatments.

### Statistical analysis

Table 2 provides an overview of the explanatory drug- and disease-specific variables and the various data sources. Drug-specific variables relate to an aspect of the drug itself, (e.g. on the type of formulation), whereas disease-specific variables describe aspects of the indication for which the drug is used (e.g. the availability of an alternative treatment). Explanatory variables were selected after a review of the literature. Relevant literature was identified by searching the MEDLINE and Embase databases. The following search terms and combinations thereof were used: orphan drug, orphan medicinal product, rare disease, rare disorder, cost, price, pricing, policies, price-setting, market access, and budget. For practical reasons, all selected articles were published in English, French or Dutch. Bibliographies of relevant articles were searched for additional references. Afterwards, variables were selected based on the availability of data related to each variable. For example, the literature indicates that severity of the diseases could be related to price; however, there is no reliable data available for rare diseases on disease severity that can be used in a regression analysis [1,5-8,10,29-36]. The common logarithms of the annual treatment costs per country were considered the outcome variables given that the original cost data were not normally distributed. Firstly, a simple linear regression analysis was performed in which all explanatory variables were individually analysed to determine a linear relation with the outcome variables. All explanatory variables, with a p-value below 0.05 in the simple linear regression analysis, were included in the multiple linear regression analysis. The multiple linear regression models were reduced stepwise by removing variables with significance levels above the 0.05 threshold. Variables with borderline significant p-values were tolerated with a view to obtaining a higher coefficient of determination (R^2^). All analyses were performed using IBM SPSS Statistics version 22.

**Table 2:** **Regression model** – **explanatory variables**

**Variable**	**Value**	**Description and source**
ONCO	0 or 1	Value 1 renders all oncology orphan drugs. Oncology orphan drugs were defined as ATC-class L (Antineoplastic and immunomodulating agents).
YAUTH	Continuous	Number of years (as an integer number) since marketing authorization of the first indication by EMA.
AvailUS	0 or 1	Value 1 renders all orphan drugs with current authorization on the US market as listed by the FDA. AvailUS is a proxy for size of the potential treatment population. [37]
MultipleIND	0 or 1	Value 1 renders all drugs with multiple orphan indications according to EMA. MultipleIND is a proxy for size of the potential treatment population.
REPUR	0 or 1	Value 1 captures all repurposed drugs. [38]
QoLimp	0 or 1	Value 1 renders all drugs for which a statistically significant improvement in quality-of-life was described in the European Public Assessment Report.
SurvImp	0 or 1	Value 1 captures all drugs for which a statistically significant improvement in survival was described in the European Public Assessment Report.
dbRCT	0 or 1	Value 1 renders all drugs for which a double blind RCT was described in the European Public Assessment Report. dbRCT is a proxy for strength of clinical trial evidence.
AltTx	0 or 1	Value 1 renders all drugs for which an alternative drug treatment is available. Supportive care, diet, palliative care, transplantation and surgical procedures were not considered as an alternative.
ultrarare	0 or 1	Value 1 renders all drugs treating ultra-rare diseases. Ultra rare diseases have a prevalence of less than 2 per 100 000 as described in the European Public Assessment Report and/or Orphanet website.
chron	0 or 1	Value 1 renders all drugs for chronic (i.e.; longer than 6months) treatments.
oral	0 or 1	Value 1 captures all oral drugs.
Cosize	0 or 1	Cosize refers to the company size of the marketing authorization holder: respectively micro and small (0) and medium and large (1) pharmaceutical companies. Company size is based on the European definition described in EU recommendation 2003/361. Data on annual sales was derived from OneSource. [39]
Weightbased	0 or 1	Value 1 captures all drugs for which the annual treatment dose is dependent on the patient’s weight or body surface area (BSA)

Additionally, the potential treatment population was calculated per orphan drug, per country and at the EU28 level by multiplying the prevalence of the indication as described in the European Public Assessment Report and/or Orphanet website with recent population data from the six countries and the EU28 as derived from Eurostat [19]. For orphan drugs with multiple indications, potential treatment populations of separate indications were summarized.

### Influence of country-specific pricing and reimbursement policies

The independent samples Mann–Whitney *U* test was used to explore whether there is an association between annual treatment costs of orphan drugs across countries with different pricing and reimbursement policies. The Mann-Whitney *U* test determines whether the overall distribution of annual treatment costs in one group (e.g. in countries with free pricing) is greater or smaller than the other (e.g. in countries with fixed pricing). Table 3 provides an overview of the different policies per country: free-market pharmaceutical pricing or price regulation (free pricing may result in higher prices for drugs); a social insurance or national health services reimbursement system; the absence or presence of a Health Technology Assessment (HTA) body; and the absence or presence of cost-effectiveness criteria when assessing orphan drug reimbursement [5]. In countries with a low GDP, budgetary restrictions can cause the exclusion of less cost-effective orphan drugs [40-43].

**Table 3:** Pricing and reimbursement policies per country

	**Belgium**	**France**	**Netherlands**	**Czech Republic**	**Italy**	**United Kingdom**
Pricing	Price negotiations	Price negotiations	Price negotiations	Fixed	Price negotiations	Free, but indirect profit control system
Reimbursement	SI	SI	SI	SI	NHS	NHS
HTA body	✓	✓	✓	✗	✗	✓
Cost-effectiveness	✗	✗	✓	✓	✗	✓

SI: Social insurance, NHS: National Health Service.

Various countries apply innovative reimbursement mechanisms, so-called ‘managed entry agreements’ (MEAs), to orphan drugs. The existence of a MEA has been documented for some orphan drugs in Belgium, England and Wales, Italy and the Netherlands by Morel *et al*. [44]. Therefore, a simple linear regression analysis was performed to determine whether the existence of a MEA for an orphan drug (0 – 1 variable) is associated with higher or lower annual treatment costs in these countries.

## Results

### Simple linear regression

At significance threshold level 0.05, ten explanatory variables appeared to play a role in the price setting of orphan drugs (Table 4). Orphan drugs which are also available in the US (1), with multiple orphan indications (2), for chronic treatments (3), for which an improvement in QoL (4) or survival (5) has been demonstrated or for ultra-rare indications (6) are correlated with higher annual treatment costs. Orphan drugs for which an alternative treatment is available (7), which are repurposed (8), which are administered orally (9), or for which the market authorization holder (MAH) is a medium- or large-sized pharmaceutical company (10) are associated with lower annual treatment costs. The Spearman test for non-parametric bivariate correlations showed a correlation coefficient (*rho*) of −0.408 (2-sized p = 0.000) between variables ‘ultra-rare indication’ and ‘alternative treatment’, and a *rho* of 0.309 (p = 0.007) between ‘oral’ and ‘chronic’, suggesting a mild correlation between these variables. Only a strong correlation (|*rho*| > 0.7) between variables implies that one of them should be omitted from the analysis.

**Table 4:** Simple linear regression analysis

	**Belgium n = 51**		**France n = 59**		**Netherlands n = 52**		**Czech Republic n = 41**		**Italy n = 28**		**United Kingdom n = 66**	
**Variable (x)**	**logBeATXcost=**	**p-value**	**logFrATXcost=**	**p-value**	**logNlATXcost=**	**p-value**	**logCzATXcost=**	**p-value**	**logItATXcost=**	**p**-**value**	**logUkATXcost=**	**p-value**
Cosize	4.422-0.214x	0.329	4.449-0.175x	0.360	4.033 + 0.258x	0.176	4.647-0.520x	0.015*	4.534-0.228x	0.477	4.505-0.115x	0.465
ONCO	4.290 + 0.007x	0.973	4.321 + 0.081x	0.677	4.000 + 0.313x	0.107	4.270 + 0.203x	0.361	4.573-0.326x	0.305	4.414 + 0.040x	0.804
YAUTH	4.584-0.042x	0.406	4.664-0.049x	0.220	4.396-0.044x	0.196	4.765-0.060x	0.267	4.519-0.006x	0.942	4.665-0.039x	0.204
AvailUS	3.837 + 0.600x	0.014*	3.829 + 0.661x	0.004*	3.967 + 0.242x	0.234	4.078 + 0.399x	0.104	3.973 + 0.543x	0.328	4.240 + 0.250x	0.189
MultipleIND	4.159 + 0.558x	0.024*	4.142 + 0.645x	0.001*	3.980 + 0.760x	0.001*	4.277 + 0.277x	0.241	4.277 + 0.595x	0.047*	4.292 + 0.455x	0.007*
REPUR	4.473-0.816x	0.001*	4.495-0.684x	0.003*	4.324-0.711x	0.000*	4.502-0.771x	0.006*	4.505-0.793x	0.303	4.558-0.545x	0.003*
QoLimp	4.239 + 0.451x	0.171	4.248 + 0.556x	0.019*	4.066 + 0.535x	0.069	4.313 + 0.427x	0.200	4.313 + 0.628x	0.052	4.368 + 0.348x	0.090*
Survimp	4.242 + 0.321x	0.274	4.344 + 0.069x	0.803	4.048 + 0.592x	0.031*	4.255 + 0.497x	0.057	4.424 + 0.350x	0.394	4.391 + 0.206x	0.303
dbRCT	4.341-0.089x	0.682	4.389-0.068x	0.724	4.265-0.278x	0.146	4.562-0.390x	0.074	4.435-0.085x	0.772	4.440-0.017x	0.915
altTX	5.000-0.883x	0.001*	4.919-0.782x	0.000*	4.471-0.436x	0.059	4.958-0.676x	0.039*	5.098-0.988x	0.000*	4.906-0.669x	0.000*
Ultrarare	4.200 + 0.303x	0.171	4.245 + 0.295x	0.132	4.132 + 0.163x	0.420	4.381 + 0.086x	0.752	4.281 + 0.353x	0.249	4.347 + 0.193x	0.249
Chronic	3.883 + 0.554x	0.021*	3.954 + 0.493x	0.040*	3.963 + 0.228x	0.289	4.310 + 0.073x	0.785	3.947 + 0.620x	0.124	4.114 + 0.414x	0.027*
Weightbased	4.185 + 0.216x	0.315	4.289 + 0.142x	0.458	4.231-0.209x	0.276	4.222 + 0.305x	0.167	4.346 + 0.351x	0.242	4.321 + 0.235x	0.143
Oral	4.563-0.500x	0.018*	4.609-0.449x	0.017*	4.154-0.040x	0.840	4.650-0.540x	0.012*	4.705-0.411x	0.156	4.623-0.400	0.011*

Explanatory variables are described in Table 2. N denotes the number of observations. All p-values marked in bold with an asterisk are significant at a 0.05 threshold level. ATXcost = annual treatment cost.

### Multiple linear regression

The multiple linear regression models showed, after stepwise reduction, significant associations between in total seven explanatory variables and the logarithm of the average annual treatment costs in different countries (Table 5). Again, repurposed orphan drugs, orally administered orphan drugs or orphan drugs for which an alternative treatment is available are associated with lower annual treatment costs. Orphan drugs with multiple orphan indications, for chronic treatments or for which an improvement in overall survival or QoL has been demonstrated, are associated with higher annual treatment costs. An average R^2^ of 0.674 was obtained, suggesting that the variables above explain around 67.5% of the variability in orphan drug annual treatment costs. Results from the Czech and Italian regression model may be underpowered, due to the smaller number of observations.

**Table 5:** Country-specific multiple linear regression models after stepwise reduction

	**Belgium R**^**2**^ **= 0.739 n = 51 Constant = 4.734**		**France R**^**2**^ **= 0.716 n = 59 Constant = 4.911**		**Netherlands R**^**2**^ **= 0.570 n = 52 Constant = 4.170**		**Czech republic R**^**2**^ **= 0.706 n = 41 Constant = 4.329**		**Italy R**^**2**^ **= 0.656 n = 28 Constant = 5.098**		**United Kingdom R**^**2**^ **= 0.661 n = 66 Constant = 4.652**	
**Variable (x)**	**Unstand. coeff.**	**p-value**	**Unstand. coeff.**	**p-value**	**Unstand. coeff.**	**p-value**	**Unstand. coeff.**	**p-value**	**Unstand. coeff.**	**p-value**	**Unstand. coeff.**	**p-value**
MultipleIND			0.504	0.001	0.570	0.010					0.312	0.028
Repurposed	−0.573	0.006	−0.548	0.002	−0.557	0.005	−0.656	0.006			−0.325	0.039
altTx	−0.478	0.026	−0.584	0.001					−0.988	0.000	−0.397	0.011
Oral	−0.637	0.000	−0.328	0.023			−0.678	0.001			−0.392	0.006
Survimp							0.443	0.035				
Chronic	0.464	0.025					0.445	0.044			0.296	0.089
ImprovQoL	0.565	0.021					0.509	0.060				

Explanatory variables are described in Table 2. N denotes the number of observations. Unstand. coeff. = unstandardized coefficients.

Finally, the independent samples Mann–Whitney *U* test showed a significantly larger potential treatment population (p = 0.034) in all countries and in the EU28 for orphan drugs with multiple orphan indications compared to orphan drugs with one orphan indication.

### Influence of country-specific pricing and reimbursement policies

No association was found between annual treatment cost of orphan drugs across countries and the pricing system (p = 0.209), the reimbursement system (p = 0.122), the availability of an HTA body (p = 0.408) or the requirement of cost-effectiveness at reimbursement (p = 0.791). Finally, the simple linear regression analysis showed no significant association between the annual treatment costs of orphan drugs and the existence of MEAs for specific orphan drugs in Belgium (p = 0.803), England and Wales (p = 0.215), Italy (p = 0.805) and the Netherlands (p = 0.071).

## Discussion

This study has identified various determinants of pricing of orphan drugs. The multiple linear regression models show significant associations between the average annual treatment cost and seven explanatory variables. Furthermore, our results seem to suggest that there are no large variations in orphan drug prices between countries in this study, in spite of differences in pricing and reimbursement policies. The annual treatment cost per patient of 18% of orphan drugs in this study exceeds €100 000.

Firstly, orphan drugs with multiple orphan indications are associated with higher prices. These results suggest that the combined prevalence is not a determining factor for price setting. Indeed, previous research showed that orphan drug prices are determined based on the prevalence of the first indication [36,45]. Launch prices for the first indication are unlikely to be reviewed following approval in other indications [46]. We calculated that orphan drugs with multiple indications have a significantly larger potential market size, although it is essential to bear in mind that no correct estimates of prevalence exist and not all patients are diagnosed and/or treated [47]. As such, the larger potential market size, combined with the higher prices for these drugs, increase the likelihood of these drugs becoming sufficiently profitable. The European regulation provides a framework for the reduction of the period of market exclusivity for orphan drugs which are sufficiently profitable, but it has never been put into practice [36,48]. There is growing concern about the increasing prices of (orphan) drug treatments [49]. For example, the median monthly price of anticancer drugs increased from US$ 1,600 in the early 1990s, to more than US$ 4,000 for anticancer drugs approved between 2000 and 2005 [50]. However, we could not confirm a correlation between the price of orphan drugs and the number of years since marketing authorization.

Secondly, repurposed orphan drugs are associated with lower prices. Rollet *et al*. also showed that the average annual treatment cost was the lowest for the category of repurposed orphan drugs [9]. It was suggested that the price of the initial common disease indication influences the price of a repurposed orphan drug. In contrast, a study in which Belgian hospital prices per defined daily dose of the medicine for the common indication versus the rare indication were compared, price differences of up to 200-fold were reported. However, this study related to a specific subset of repurposed drugs for which the effectiveness evidence was already established prior to the orphan designation [38].

Under the soon-to-be introduced value-based pricing (VBP) system by NICE in the UK, all new medicines will be priced according to their value. A study suggests that diseases for which no other treatments are available represent an unmet need and should be prioritized under the VBP system [51]. Indeed, unmet need (i.e. the absence of alternative treatments), therapeutic benefit (i.e. QoL or survival improvement) and therapeutic value (i.e. high quality of clinical trial data, ease of administration) could warrant high (er) prices. Our results show that the availability of an alternative drug treatment is associated with lower prices for orphan drugs. In the past, only a non-significant trend has been reported between the number of available alternatives for an orphan drug and its price [11]. Also, orphan drugs for which an improvement in survival and/or QoL has been demonstrated, are associated with higher prices. Roos *et al*. claim that the therapeutic benefit of an orphan drug does not correlate with its price [6]. Due to difficulties in generating evidence to demonstrate therapeutic benefit, this information is often lacking or incomplete when the price is set. We found no association between the existence of a double blind RCT and the price of an orphan drug. A more in-depth analysis of clinical trial data is required. From the patients’ point of view, the ease of administration of an oral drug constitutes a considerable therapeutic value as some intravenous or inhalation therapies can be time-consuming. Our results correlate oral orphan drugs with lower prices and chronic treatments with higher prices. Popular media claimed that the most expensive drugs are injectable drugs whose dosing varies by weight [52]. Our results indicate that indeed, oral orphan drugs are cheaper. However, we could not confirm a correlation between weight-dependent dosing and price.

Finally, we found no correlation between different pricing and reimbursement policies and the annual treatment cost of orphan drugs across countries in this study. However, as countries in this study likely have a similar ability to pay for orphan drugs, we cannot conclude that there is a trend towards uniform pricing of orphan drugs in Europe. Additionally, many countries have special regulations governing orphan drugs market [40]. For example, in France, Italy and the Netherlands, there are specific policy measures and incentives to promote research and development of orphan drugs [20]. In England, some orphan drugs are funded by the national Cancer Drug Fund from the National Health Services [53]. In the Netherlands, hospitals are financially supported for prescribing orphan drugs through the Dutch Policy Rule for Expensive Hospital and Orphan Drugs [54]. A differential pricing system, in which price is dependent on a country’s GDP, could enhance equal access to orphan drugs [29]. However, the success of differential pricing depends on the ability to limit parallel export between countries [55]. Prices obtained after country-specific price negotiations or in MEAs are not captured by this study. Higher prices for orphan drugs do not increase the likelihood of a MEA.

This study provides insight into the interplay of orphan drugs pricing and various drug and disease specific explanatory variables. The variables in the multiple linear regression models explain on average 67.5% of the variability in orphan drug prices. The effects of each variable were considered independently (i.e. no interactions between variables were added to the model), thereby reducing the risk of overfitting. Due to the availability of data, we used a mix of public prices and ex-factory prices, although results tended to be consisted across countries. Final prices are determined based on confidential negotiations with governments and are also influenced by rebates and discounts negotiated with insurers, wholesalers and hospital pharmacies [9]. Negotiated prices are confidential and are likely to show more variability across countries. We were unable to determine whether publicly available prices account for rebates and/or are the result of negotiations. The transformation of some variables to binary variables constitute both a strength and a weakness, as it increases the likelihood of obtaining significant results, but may also oversimplify the analysis. Finally, annual treatment dosages were calculated for “standard” patients without accounting for lower adherence in daily life, lower dosages for liver- and/or renal-impaired patients or other patient-specific dose adjustments. Weights of patients obtained from clinical trial data can possibly be skewed due to the trial inclusion criteria.

## Conclusion

This study has shown that prices of orphan drugs are influenced by factors such as the availability of an alternative drug treatment, repurposing, the length of treatment, the administration route, the presence of multiple indications and the impact on overall survival and QoL. Yet, a lot of vagueness still surrounds the orphan drug pricing mechanism. Current debate about the affordability of high-priced orphan drugs and the impact on accessibility highlights the need for more transparency around orphan drug price setting. Within the context of value-based pricing decisions, a Transparent Value Framework (TVF) has been proposed with a set of indicative assessment criteria [56]. The outcome of a TVF assessment could be included as a factor in pricing negotiations. Unfortunately, country-specific price negotiations may also add to the complexity of orphan drug pricing.

## Abbreviations

SmPC: Summary of product characteristics; BSA: Body surface area; EPAR: European public assessment report; OS: Overall survival; HTA: Health technology assessment; MEAs: Managed entry agreements; MAH: Marketing authorization holder; VBP: Value-based pricing; TVF: Transparent value framework.

## Competing interests

The authors have no conflicts of interest that are directly relevant to the content of this manuscript. Thomas Morel is an employee of UCB. The authors would like to thank Miss Lenka Pavlacka for her assistance in collecting orphan drug prices in the Czech Republic.

## Authors’ contributions

The work presented was carried out in collaboration between all authors. EP, TM, and SS were involved in the initiation of the project and the design of the methods. EP and TM participated in the data collection. EP analysed the data and interpreted the results. EP, TM, DC and SS discussed the results. EP and SS wrote the draft paper. All authors revised the draft paper. All authors read and approved the final manuscript.
